# Transcriptomic dissection of termite gut microbiota following entomopathogenic fungal infection

**DOI:** 10.3389/fphys.2023.1194370

**Published:** 2023-04-21

**Authors:** Ya-ling Tang, Yun-hui Kong, Sheng Qin, Austin Merchant, Ji-zhe Shi, Xu-guo Zhou, Mu-wang Li, Qian Wang

**Affiliations:** ^1^ Jiangsu Key Laboratory of Sericultural Biology and Biotechnology, School of Biotechnology, Jiangsu University of Science and Technology, Zhenjiang, Jiangsu Province, China; ^2^ Shanghai First Maternity and Infant Hospital, Tongji University School of Medicine, Shanghai, China; ^3^ Key Laboratory of Silkworm and Mulberry Genetic Improvement, Ministry of Agriculture and Rural Affairs, Sericultural Research Institute, Chinese Academy of Agricultural Science, Zhenjiang, Jiangsu Province, China; ^4^ Department of Entomology, University of Kentucky, Lexington, KY, United States

**Keywords:** termite, odontotermes formosanus, metarhizium robertsii, *de novo* assembly, innate immunity, gut microbiota

## Abstract

Termites are social insects that live in the soil or in decaying wood, where exposure to pathogens should be common. However, these pathogens rarely cause mortality in established colonies. In addition to social immunity, the gut symbionts of termites are expected to assist in protecting their hosts, though the specific contributions are unclear. In this study, we examined this hypothesis in *Odontotermes formosanus*, a fungus-growing termite in the family *Termitidae*, by 1) disrupting its gut microbiota with the antibiotic kanamycin, 2) challenging *O. formosanus* with the entomopathogenic fungus *Metarhizium robertsii*, and finally 3) sequencing the resultant gut transcriptomes. As a result, 142531 transcripts and 73608 unigenes were obtained, and unigenes were annotated following NR, NT, KO, Swiss-Prot, PFAM, GO, and KOG databases. Among them, a total of 3,814 differentially expressed genes (DEGs) were identified between *M. robertsii* infected termites with or without antibiotics treatment. Given the lack of annotated genes in *O. formosanus* transcriptomes, we examined the expression profiles of the top 20 most significantly differentially expressed genes using qRT-PCR. Several of these genes, including APOA2, Calpain-5, and Hsp70, were downregulated in termites exposed to both antibiotics and pathogen but upregulated in those exposed only to the pathogen, suggesting that gut microbiota might buffer/facilitate their hosts against infection by finetuning physiological and biochemical processes, including innate immunity, protein folding, and ATP synthesis. Overall, our combined results imply that stabilization of gut microbiota can assist termites in maintaining physiological and biochemical homeostasis when foreign pathogenic fungi invade.

## 1 Introduction

Microbial symbionts inhabit the guts of many insects, including beetles, silkworms, fruit flies, and termites (Cheng et al., 2017; Garcia-Robles et al., 2020; Li et al., 2021; Vikram et al., 2021). Gut microbiota can be seen as a virtual organ whose existence is indispensable to the life of the host (Gravitz, 2012). Termites possess a wide variety of gut symbionts, including but not limited to bacteria, protists, and fungi, which play many important roles in physiological and biochemical processes such as social alarm, hygienic responses, metabolic capacity, and immunity (Xiong, 2022).

Termites are social insects that live in colonies consisting of three basic caste types: workers, soldiers, and reproductives (He et al., 2018). Because they often live in the soil or in decaying wood, pathogens found in these substrates pose a significant threat to termite colonies (Peterson and Scharf, 2016a). How termites contend with these threats, through mechanisms such as individual and social immunity, has gradually become a new hot spot for research (Avulova and Rosengaus, 2011; Cremer et al., 2018; Hong et al., 2018).

The defensive function of gut microbiota against invading pathogens has been studied in other insects (Zhou et al., 2020; Deng et al., 2022). *Serratia* and other symbionts render mosquitoes resistant to plasmodium infection through activation of the host immune system or by secreting antimalarial enzymes (Xi et al., 2008; Ramirez et al., 2012; Bai et al., 2019; Gao et al., 2021). In *Drosophila*, gut microbiota help flies fight pathogens by mediating the renewal of intestinal epithelial cells, and expression of host immune-related genes is altered after the removal of intestinal microorganisms (Buchon et al., 2009; Douglas, 2018).

The lower termites possess particularly robust defenses against fungal pathogens. Individual workers may ingest a large number of fungal spores while grooming nestmates, which become inactive in the gut and do not germinate (Chouvenc et al., 2009). An antimicrobial peptide with antifungal activity, Termicin, was discovered, in addition to synthesis by gut protozoa of the multifunctional *β*-1,3-glucanases, which break down fungal cell walls (Rosengaus et al., 2014). Similarly, intestinal symbionts protect lower termites from entomopathogenic fungi by producing antifungal compounds and collaborating with host endogenous compounds containing antifungal peptides or enzymes (Peterson and Scharf, 2016b).

In *Odontotermes formosanus*, a higher termite, only bacterial symbionts are found in the gut (Liu et al., 2013; Hu et al., 2019). Comparably less research has been done on the contributions of bacterial symbionts to termite immunity relative to protists (Peterson and Scharf, 2016a). Previous research on termite immunity has revealed differences in species diversity and relative abundance of the gut microbiota between healthy and pathogen-infected termites (Liu et al., 2015). In addition, the composition and diversity of intestinal symbionts influences gene expression in the termite gut (Xi et al., 2008; Gao et al., 2021). Therefore, it is important to investigate the impacts of bacterial symbionts on termite physiology and immunity.

In this study, we changed the abundance and load of gut microbiota in the hindgut of the higher termite, *O. formosanus,* which is widely distributed in China, with a broad-spectrum antibiotic, kanamycin, a bacterial antibiotic which binds to the bacterial ribosome 30S subunit to inhibit protein synthesis (Shu et al., 2011). In our previous 16s rDNA sequencing experiment, we found kanamycin significantly impacted intestinal composition, especially *Firmicutes* and *Spirochete*. Then, we infected termites with a pathogenic fungus, *Metarhizium robertsii*, and measured the expression changes of genes related to immunity and energy metabolism in intestinal tissue. Analyzing the effects of specific gene expression changes following immune challenge contributes to further understanding of the symbiotic relationship between gut microbiota and their termite hosts and can also provide reference and inspiration for related research in other insects.

## 2 Materials and methods

### 2.1 Experimental insects and setup

*O. formosanus* colonies were collected from Lion Mountain (Huazhong Agricultural University, Wuhan, Hubei, China) in July 2020. Termites were transferred from pieces of rotting wood to 9 cm diameter Petri dishes with filter paper and placed in darkness for 24 h to stabilize the community. Afterwards, termites were maintained in 9 cm diameter Petri dish in 24-h darkness at 25°C ± 1°C and were fed with filter paper soaked with sterile water that was changed once every 2 days. *M. robertsii* ARSEF#2575 was obtained from the Institute of Plant Physiology and Ecology, Chinese Academy of Sciences, Shanghai. *M. robertsii* spores were grown on Potato Dextrose Agar medium (PDA) (200 g potato, 20 g glucose, 15–20 g agar, 1000 mL distilled water) and washed with 0.1% Tween 80 solution. Spore concentration was measured with a hemocytometer, then spores were stored at 4°C as a spore suspension. The concentration of antibiotics used was determined by previous research (Peterson et al., 2015).

Before fungal infestation, we separated termite colonies into four experimental groups (control groups/CT: treated with sterilized water; Kana groups/AT: treated with 5% kanamycin; Kana_infected groups/IAA: treated with 10^8^ spores/mL suspension at 48 h time point after treatment with 5% kanamycin; Infected groups/MI: treated with 10^8^ spores/mL suspension at 48 h time point), with every group consisting of three biological replications containing 30 termites each. 5% kanamycin means that the ratio of powder to distilled water is 1:20. Termites were reared on a filter paper soaked with 1 mL of the corresponding solution for their treatment group. Before dissecting, all termite samples were placed on ice and 75% ethanol was used to remove any surface microorganisms. We use anatomical forceps to tear open the abdominal epidermis of termites under a posture microscope, pull out the intestine, and ensure its integrity then transferred to an empty Eppendorf tube. The entire process is immersed in 1× sterile PBS (Phosphate Buffer Solution). All gut samples were stored at −80°C until RNA extraction.

### 2.2 RNA extraction

All samples were homogenized in Trizol (Ambion/Invitrogen, United States) following the manufacturer’s protocol, purified by isopropanol precipitation, and dissolved in DEPC water. Each treatment contains 30 termite samples and sets up 3 biological replicates. RNA concentration was measured using 1% agarose gel electrophoresis and a Nano-Drop 2000 spectrophotometer. RNA completion was measured using an Agilent 2,100 bioanalyzer. Then, RNA products were stored in a −80°C cryogenic refrigerator.

### 2.3 Library preparation and sequencing

Library preparation and sequencing were performed by Novogene (Tianjin, China) on an Illumina NovaSeq 6,000 (Illumina Inc., San Diego, United States) platform. mRNA was isolated using Oligo (dT) magnetic beads (Thermo Fisher Scientific, Waltham, Mass., United States). Subsequently, the obtained mRNA was fragmented using NEB Fragmentation Buffer (New England Biolabs (Beijing) LTD., Beijing, China). Using fragmented mRNA as a template and random oligonucleotides as primers, the first strand of cDNA was synthesized using the M-MuLV reverse transcriptase system, and the RNA strand was degraded using RNase H (TAKARA Bio, Beijing, China). In the DNA polymerase I system, dNTPs (TAKARA Bio, Beijing, China) were used as primers to synthesize the second strand of cDNA. The purified double-stranded cDNA underwent end-repair, A-tailing, and ligation of sequencing adapters. AMPure XP beads (Beckman Coulter, Shanghai, China) were used to screen 250-300bp cDNAs, then PCR amplification was performed and AMPure XP beads were used again to purify the PCR products, which constituted the complete library. After the library was constructed, preliminary quantification was carried out using a Qubit2.0 Fluorometer (Thermo Fisher Scientific, Waltham, Mass., United States), then, library concentration was diluted to 1.5 ng/µL. Next, insert size was assessed with an Agilent 2,100 bioanalyzer (Agilent Technologies, Beijing, China). The raw data were submitted to NCBI (National Center for Biotechnology Information) databases (Accession numbers: PRJNA926100).

### 2.4 De novo assembly and functional annotation

Adapter and low-quality reads were filtered by Fastp (version 0.19.7) (Chen et al., 2018) with default parameters in order to obtain clean data. Clean data was assembled to contigs using Trinity (v2.4.0) software with min_kmer_cov:3 and other default parameters (Grabherr et al., 2011). Based on Trinity splicing, transcripts were aggregated into clusters according to shared reads between transcripts by Corset (v4.6) (Davidson and Oshlack, 2014) with default parameters. Annotation to the NCBI Non-redundant Protein Sequences (NR), Nucleotide (NT), Eukaryotic Orthologous Groups (KOG), Swiss-Prot, Protein Families (PFAM), Gene Ontology (GO), and KEGG Orthology (KO) databases was carried out using BLASTX for all assembled unigenes (E-value≤1e-5) (Altschul et al., 1997; Moriya et al., 2007; Kanehisa et al., 2008; El-Gebali et al., 2019).

### 2.5 Differential gene expression analysis

The transcripts were assembled using Trinity as reference sequence and clean data were mapped to the reference sequence using RSEM and bowtie2 with default parameters (Li and Dewey, 2011). Then, read counts were obtained by calculating the bowtie2 result and the FPKM value of each gene was derived. Differences in gene expression were analyzed using the DESeq2 R package (1.6.3) with default parameters. Finally, |log2FoldChange| > 1 and P-adj < 0.05 were set as standards to identify differentially expressed genes (DEGs) (Wang et al., 2010; Love et al., 2014).

### 2.6 KEGG and GO enrichment of DEGs

Enriched GO terms among differentially expressed genes were identified using GOseq (Young et al., 2010) and KOBAS3.0 was used to analyze the statistical enrichment of DEGs among KEGG pathways (Mao et al., 2005).

### 2.7 Quantitative real-time PCR (qRT-PCR)

A total of 1.0 μg of RNA was reverse-transcribed *in vitro* using a PrimeScript™ RT reagent kit, following the manufacturer’s instructions. The cDNA products were used as a template for real-time qPCR. RT-qPCR was used to detect gene expression levels. The primers used in this study were designed by NCBI Primer-BLAST software (https://www.ncbi.nlm.nih.gov/tools/primer-blast/) and are shown in Table 1. The reaction mixtures were prepared using a NovoStart^®^ SYBR qPCR SuperMix Plus kit (Novoprotein Technology Ltd., China), following the manufacturer’s instructions. A 10 μL qPCR reaction system was used, including 5 μL of 2 × NovoStart^®^ SYBR qPCR SuperMix Plus, 0.5 μL of upstream and downstream primers, 1.5 μL of the template, and 2.5 μL of ddH2O. The reactions were performed on a LightCycler^®^ 96 System (Roche, Basel, Switzerland). The following qPCR protocol was used: 1 cycle at 95°C for 5 min, followed by 40 cycles at 95°C for 20 s, and 54°C for 40 s. The 2^−ΔΔCT^ method was adopted to calculate relative gene expression levels. Each group was repeated three times. β-actin was used as the internal reference gene (Supplementary Table S4).

**TABLE 1:** Summary of *de novo* assembled transcriptomes.

Samples	Raw reads	Raw bases (G)	Clean reads	Clean bases (G)	Error rate (%)	Q20 (%)^1^	Q30 (%)^1^	GC (%)
**AT-1**	29548065	8.9	29066618	8.7	0.03	97.03	91.93	37.25
**AT-2**	27438745	8.2	27008031	8.1	0.03	97.09	91.95	38.28
**AT-3**	31207335	9.4	30672455	9.2	0.03	97.31	92.41	38.64
**MI-1**	28509413	8.6	27855614	8.4	0.03	97.17	92.19	36.94
**MI-2**	28951294	8.7	28588750	8.6	0.03	97.18	92.19	38.90
**MI-3**	25660698	7.7	25350768	7.6	0.03	97.17	92.20	37.51
**CT-1**	29767000	8.9	29024356	8.7	0.03	97.72	93.21	36.46
**CT-2**	30811690	9.2	29749815	8.9	0.03	97.86	93.65	38.92
**CT-3**	32076085	9.6	31439083	9.4	0.03	96.85	91.21	36.44
**IAA-1**	28976224	8.7	27930028	8.4	0.03	97.03	91.77	39.59
**IAA-2**	3,3562021	10.1	32117335	9.6	0.03	95.99	89.79	40.71
**IAA-3**	35311566	10.6	34005244	10.2	0.03	96.11	89.93	40.32

^1^ Q20/Q30 stands for the percent of bases with quality score [-10 * lg (error rate)] more than 20 and 30 (indicating error rates of 1% and 1‰, respectively).

## 3 Results

### 3.1 Kanamycin accelerates termite death by compromising its gut microbiota

**FIGURE 1:**
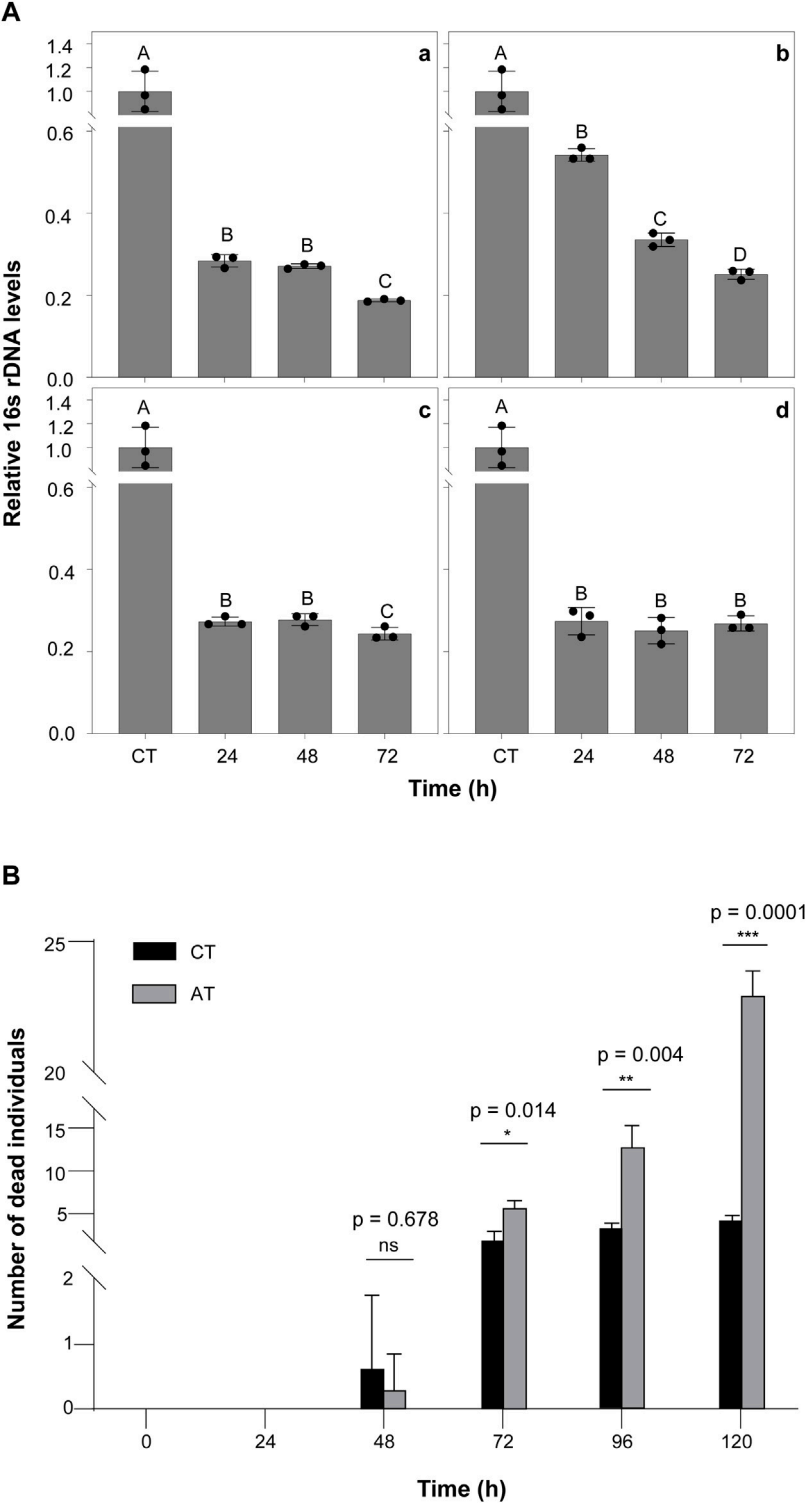
The Effect of Antibiotics on Termites **(A)** The effects of antibiotic treatment on bacterial symbiont abundance in the termite gut. The qPCR results for total bacteria in the guts of termites treated with 5% Kanamycin (a), 5% Ampicillin (b), 5% Streptomycin (c), and 2.5% Tetracycline (d). β-actin was used to normalize the data, which are shown as mean ± standard error; the mean was obtained from three independent replications. The 2^−ΔΔCT^ method was used to calculate the relative expression level. Statistical differences between time points, determined with an unpaired *t*-test, are shown via GraphPad Prism8. Different letters indicate significant differences (B means *p* < 0.0001, C means *p* < 0.05). **(B)** Number of dead termite individuals fed with 5% kanamycin.AT termites were fed with 5% kanamycin, while CT termites were fed with sterile water. The experiments were performed in three biological replicates. Kanamycin significantly increased mortality compared with the control group after 72 h (*p* < 0.05). The experiments were performed in three biological replicates (n = 30 per replicate). Statistical differences between treatment at every time points, determined with an paired *t*-test, are shown via GraphPad Prism8.

To verify which antibiotics can play a significant role in removing termite intestinal microorganisms, we fed termites with four different common antibiotics and measured the change in total bacteria abundance at multiple time points (0, 24, 48, and 72 h) using qRT-PCR (primers shown in Table 1, termite *β-actin* as reference gene). The total bacterial changes in the guts of termites exposed to four different antibiotic treatments are shown in Figure 1. The largest change was observed in the kanamycin-treated individuals, particularly at the 24 h time point (*p* < 0.0001) (Figure 1A a). Based on the results of this assay, kanamycin was ultimately selected as the antibiotic to be used in the following experiments.

Moreover, there was no significant difference in the survival rate of kanamycin treated termites at 48 h compared to controls (Figure 1B). At 72 h, mortality was significantly higher in the kanamycin-treated group (*p* < 0.0001). This result suggests that termites remain in a healthy state of life for at least 48 h after intestinal microorganism inhibition. Therefore, we chose to treat termites with antibiotics for 48 h before *M.robertsii* infection in subsequent experiments.

### 3.2 Transcriptome sequencing, assembly, and annotation

In this study, a total of 108.6 Gb of raw data were obtained from Illumina sequencing. After quality control, a total of 105.8 Gb of clean data was used for further analysis (Table 1). There were 142531 transcripts and 73608 Unigenes obtained by Trinity; their N50 length was 3,655 and 2746bp, respectively (Supplementary Tables S1,S2).

All Unigenes were annotated to NR, NT, KO, Swiss-prot, PFAM, GO, and KOG databases by BLASTx. 52.98% of unigenes were annotated to at least one database. The proportion of unigenes annotated to each of the seven databases was 37.72, 21.94, 13.01, 22.2, 31.31, 31.31, and 11.67%, respectively (Supplementary Table S3).

Annotated genes were placed into one of three groups based on GO classification: BP (biological process), CC (cellular component), or MF (molecular function) (Supplementary Figure S1). Among BP genes, cellular process and metabolic process were the most common classifications. Among CC genes, cellular anatomical entity, intracellular, and protein-containing complex were the most common classifications. Among MF genes, binding and catalytic activity were the most common classifications.

In total, 8,594 genes were annotated to the KOG database across 26 functional categories (Supplementary Figure S2). Over 13% of genes were annotated to the “General function prediction only” category, followed by “Posttranscriptional modification, protein turnover, chaperones” (13.25%), “Translation, ribosomal structure and biogenesis” (11.74%), and “Signal transduction mechanisms” (11.15%). Categories with the fewest annotations included “Extracellular structures” (0.08%), “Defense mechanisms” (0.06%), “Nuclear structure” (0.04%) and “Unnamed protein” (0.002%).

**FIGURE 2:**
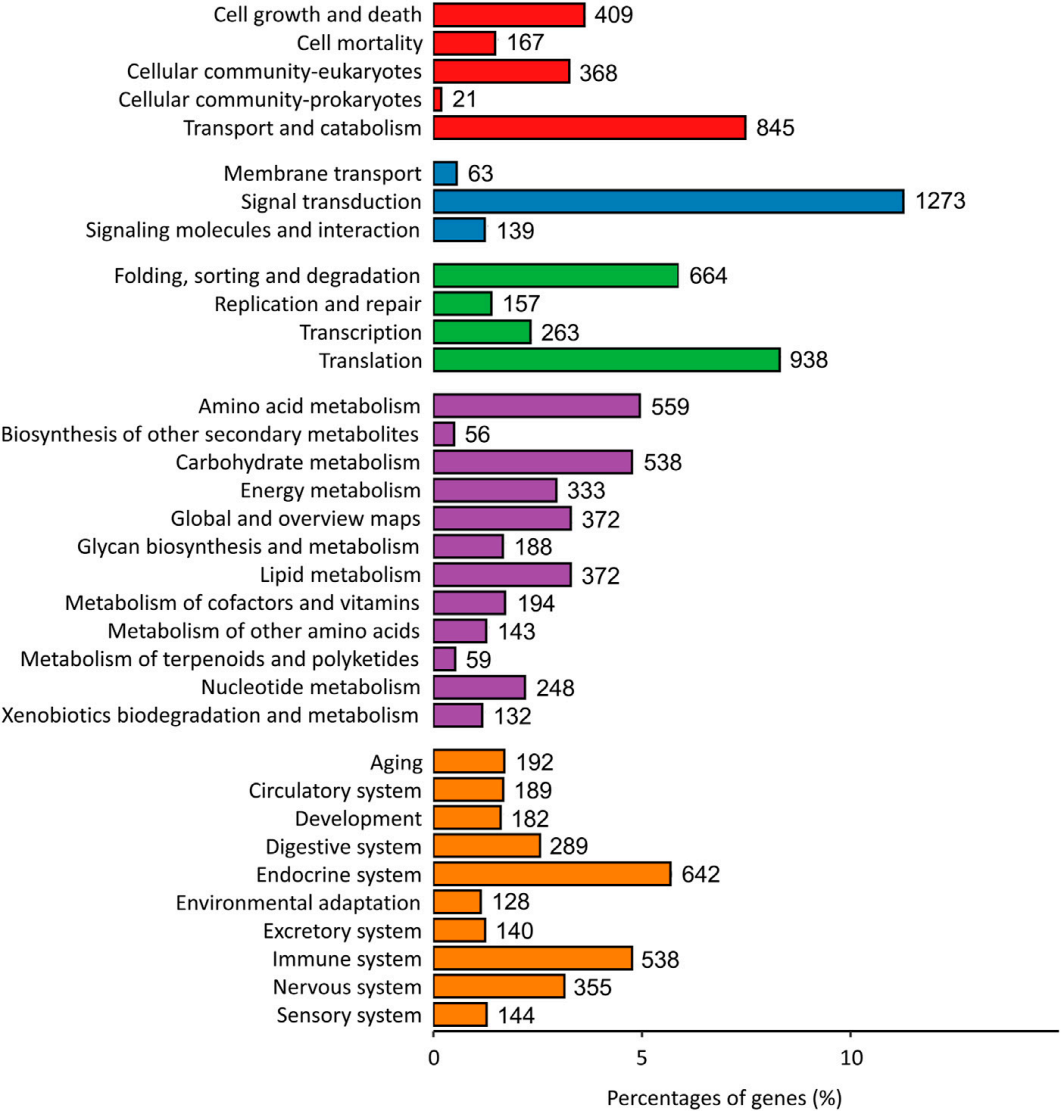
KEGG metabolic pathway classification. Annotated genes were assigned into one of five categories based on predicted function (Red bar: Cellular Processes; Blue bar: Environmental Information Processing; Green bar: Genetic Information Processing; Purple bar: Metabolism; Orange bar: Organismal Systems).

A total of 11300 genes were annotated to the KEGG pathway, distributed among 291 unique KEGG pathways. The highest number of genes were annotated to the “Cellular Processes” (1810, 16.01%), “Environmental Information Processing” (1475, 13.05%), “Genetic Information Processing” (2022, 17.89%), “Metabolism” (3,062, 27.10%) and “Organismal Systems” pathways (2,799, 24.77%) (Figure 2). Additionally, 538 genes (4.76%) were assigned to the immune system.

### 3.3 Identification of DEGs following antibiotic treatment and/or pathogen exposure

**FIGURE 3:**
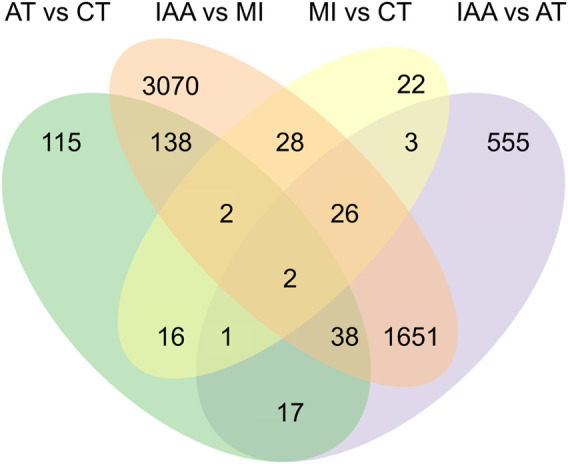
Venn diagram of DEGs among four pair-wise comparison groups.

To identify the immune effect of the inhibition of gut microorganisms on termites exposed to entomopathogenic fungi, we used DESeq2 software to analyze differences in gut gene expression between termites from four different treatment groups. A corrected *p*-value of 0.05 and log2fold-change of ±1 was set as thresholds for significant differential expression. Overall, 43 unigenes were upregulated and 57 were downregulated in MI compared to CT termites; 2,204 were upregulated, and 1610 were downregulated in IAA compared to CT termites; 2,711 were upregulated and 2,244 were downregulated in IAA compared to MI termites; 1160 were upregulated and 1133 were downregulated in IAA compared to AT termites; and 125 were upregulated and 204 were downregulated in AT compared to MI termites (Supplementary Figure S3). When termites are infected by *M. robertsii*, their gut microbiota can adapt to the external environment to a certain extent. Similarly, following antibiotics treatment, the impact on uninfected termites is smaller. A total of 22 DEGs were identified between the MI: CT group, 115 between the AT: CT group, 555 between the IAA:AT group, and 3,070 between the IAA:MI group (Figure 3).

### 3.4 Enrichment analysis of DEGs reveals the existence of gut symbionts that maintain termite intestinal stability during fungal invasion

**FIGURE 4:**
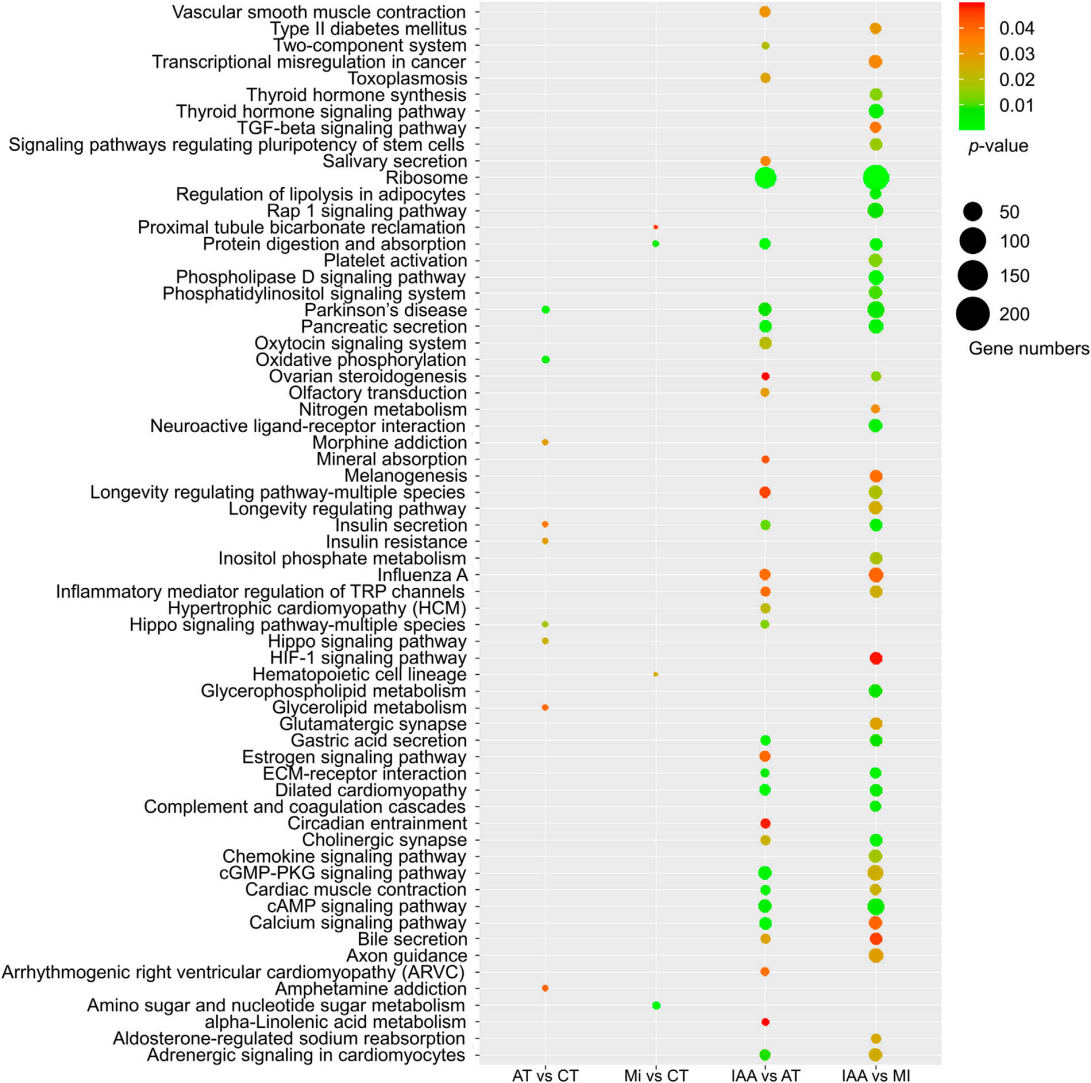
KEGG enrichment of DEGs identified in each comparison group. The size of the dot indicates the number of genes, and the *p*-value was derived by hypergeometric tests.

After identifying the numbers of DEGs among different pairs of the four treatment groups, we also examined the placement of these DEGs among KEGG pathways (Figure 4).

When we examined DEGs identified in the AT:CT group, it was found that most metabolic pathways did not change in kanamycin treating 48 h, indicating that antibiotic treatment had no negative effect on gene expression or the physiological status of the termite intestinal tract itself. Examination of DEGs identified in the MI:CT group also yielded a low number of metabolic pathways showing change despite infection by *M. robertsii*. DEGs identified in the IAA:AT and IAA:MI groups suggest significant changes in several metabolic pathways, including the cAMP and calcium signaling pathways. In addition, MAPK signaling pathway were enriched in IAA: CT groups by KEGG enrichment (Supplementary Figure S4A). These results demonstrate that the presence of intestinal microorganisms can alleviate the metabolic effect of fungal invasion to a certain extent.

**FIGURE 5:**
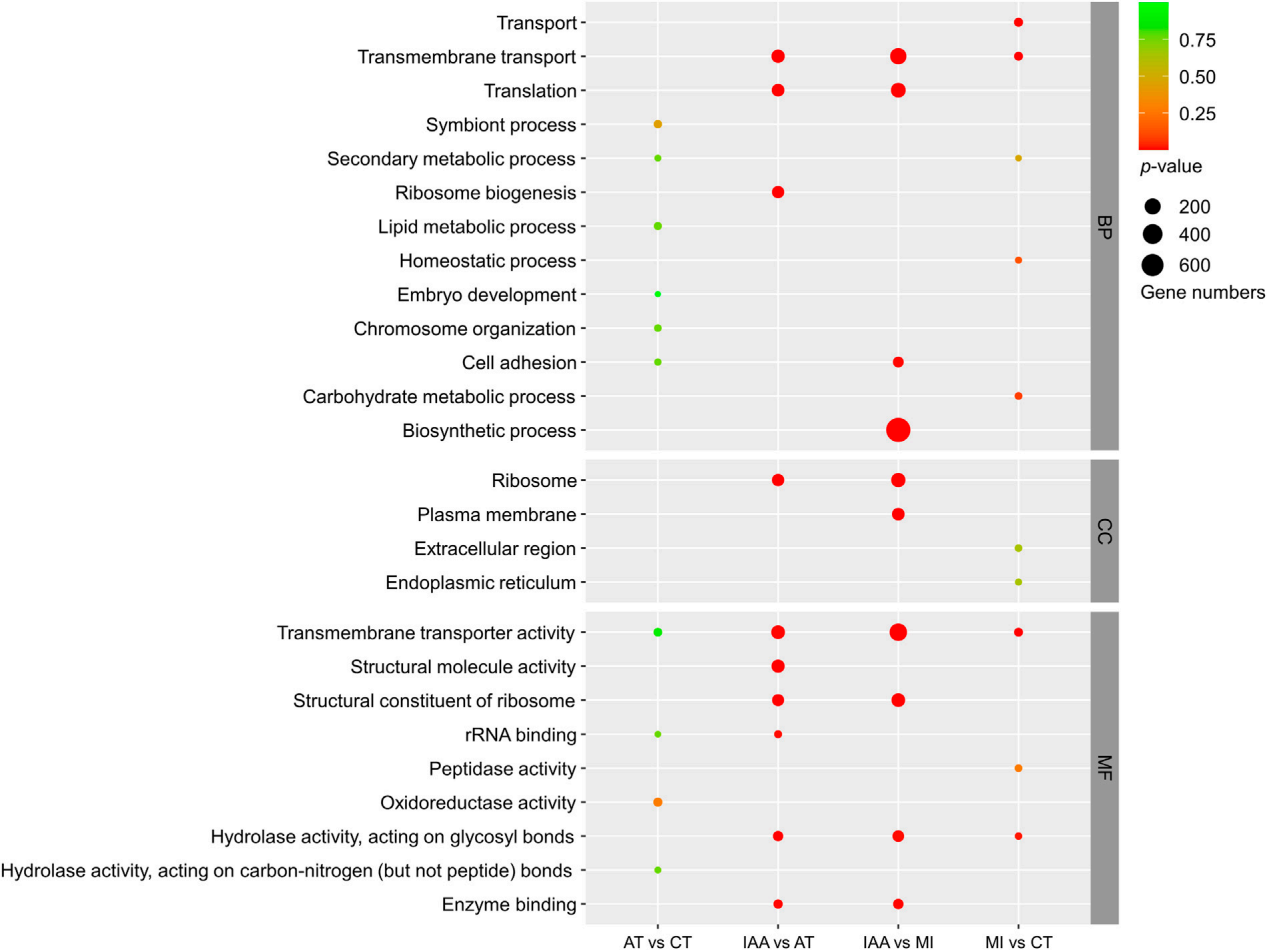
GO enrichment of DEGs identified in each comparison group. The size of the dot indicates the number of genes, and the *p*-value was derived by hypergeometric tests. BP: Biological Process; CC: Cellular Component; MF: Molecular Function.

The GO classification of DEGs identified in each comparison group was also examined (Figure 5). DEGs identified in the AT:CT and MI:CT comparisons showed minimal changes within the CC and MF categories. DEGs placed in the BP category mostly involved membrane-related reactions. DEGs identified in the IAA:AT and IAA:MI comparison groups were spread between all three GO categories.

The results of KEGG and GO enrichment indicate that a change in intestinal symbionts alters host gene expression in the gut and causes the transformation of many metabolic pathways. However, unexpectedly, the main enriched pathways were not directly related to immunity, but indirect pathways such as signal transduction, indicating that gut microorganisms may affect host immunity through other forms of regulation.

### 3.5 qRT-qPCR analysis

**FIGURE 6:**
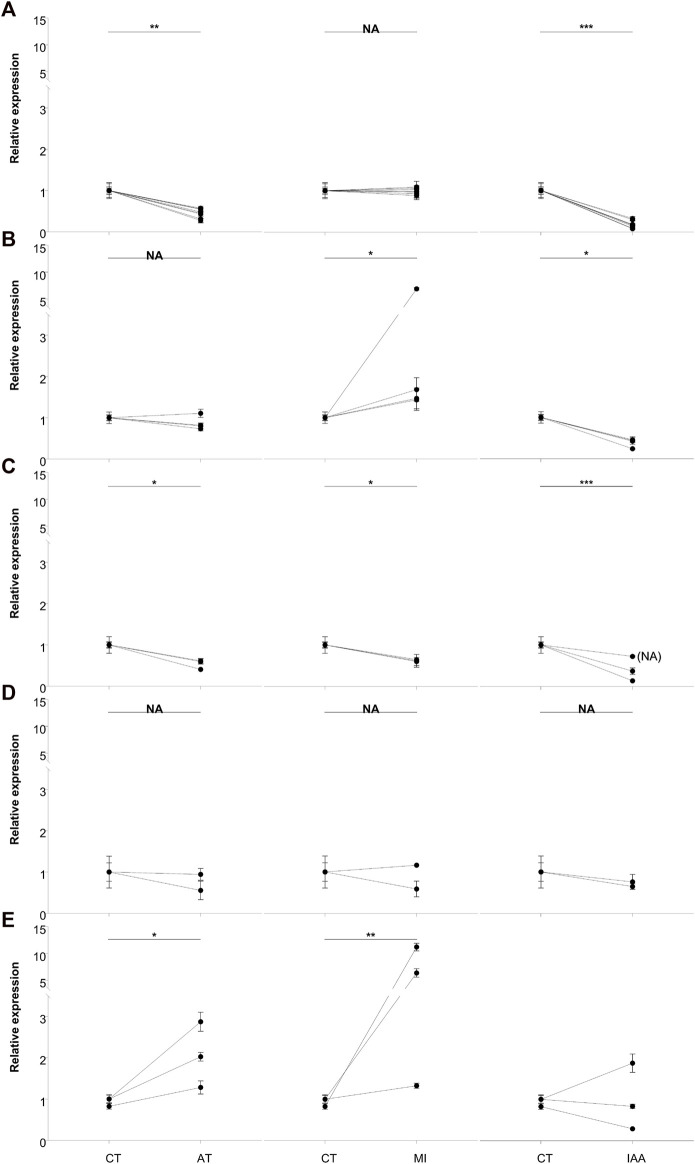
qRT-PCR analysis of the top 20 most significantly expressed genes. Based on the expression profiles, DEGs were grouped as flow: **(A)** down-regulated in AT and IAA; **(B)** upregulated in MI but down-regulated in IAA; **(C)** down-regulated in all groups; **(D)** no changed in all groups and **(E)** the remained genes. β-actin was used to normalize the data, which are shown as mean ± standard error; the mean was obtained from three independent repeats. The 2^−ΔΔCT^ method was adopted to calculate the relative expression level. The statistical difference relative to the control (CT) group, determined with an unpaired *t*-test, is shown via GraphPad Prism8. Asterisks indicates statistically significant differences (* = *p* < 0.05; ** = *p* < 0.01; *** = *p* < 0.001). (CT: treated with sterilized water as control treatment; AT: treated with 5% kanamycin antibiotic; MI: treated with 108/mL *M. robertsii* conidia suspension at 48-h; IAA: treated with 5% kanamycin, then treated with 108/mL *M. robertsii* conidia suspension at 48 h).

To further evaluate the DEGs identified in the transcriptome and better explain the role of intestinal microorganisms in termite immunity, we carried out real-time fluorescent quantitative PCR validation of the top 20 genes showing the most significant changes in expression among our comparison groups (Figure 6). We chose β-actin as the reference gene for validation. The primers used are listed in Supplementary Table S4 and Blast results are shown in Supplementary Table S5.

Gene expression patterns were divided into five groups. Eight genes showed lower expression in the AT and IAA treatments than in the MI treatment, while expression in the CT and MI treatments was relatively equal (Figure 6A). These eight genes are mainly involved in the process of reverse transcription and cell division. The second group of genes, four in total, showed upregulation in the MI treatment, downregulation in the IAA treatment, and no change in the AT treatment (Figure 6B). These genes included *ApoA2*, *Cal-5-like*, and *Hap*. The third group of genes, three in total, were downregulated in all non-CT treatments and included genes involved in sodium and potassium ion transport (Figure 6C). The change in the internal environment of termites treated with antibiotics and/or pathogens or the influence of external stimuli may inhibit this process. Next, the gene *chitinase5* mainly functions as a chitinase and participates in the decomposition of β-1,4-glucan (Figure 6D), which is the main component of plant cellulose, indicating that the antibiotic treatment does not affect the ability of termites to degrade cellulose at this time point. In addition to the genes that cannot be annotated, in these three genes, *hsp70* showed that all the treatment groups were upregulated. But *ATP-syn* expressed in MI was higher than CT or AT and low expressed in the IAA group.

## 4 Discussion

Various symbionts inhabit the intestinal tracts of termites, and these symbionts play an indispensable role in the lives of their hosts, aiding in nutrient supply, digestion, nitrogen metabolism, and more (Liu et al., 2013; Peterson and Scharf, 2016a). However, the role of intestinal microorganisms in the immunity of their termite hosts, such as when encountering a fungal pathogen, is unclear (Peterson and Scharf, 2016b). We exposed *O. formosanus* termites to antibiotics, pathogenic *M. robertsii*, or both in sequence, and then measured gut gene expression. Termites exposed to antibiotics followed by a fungal pathogen showed significantly larger changes in gene expression than those exposed only to one or the other (Figure 3), suggesting that gut symbionts can buffer their hosts against foreign pathogens.

This relationship between gut microbiota and immunity has been demonstrated previously in the lower termite *Reticulitermes flavipes*, which shows greatly increased sensitivity to the fungal pathogen *Beauveria bassiana* following antibiotic treatment (Peterson and Scharf, 2016b). A similar relationship is observed in mosquitoes, which possess midgut microorganisms that mediate host resistance to foreign pathogens, including plasmodium (Dong et al., 2009; Aida et al., 2013; Gao et al., 2021), viruses (Xi et al., 2008), and pathogenic fungi (Wei et al., 2017). Most of these symbionts directly affect the gene expression of the Toll or Imd immune pathway of the host.

The results of our GO and KEGG enrichment analyses indicated that identified DEGs were not involved in direct immune pathways, but rather in signaling pathways, including the calcium signaling pathway (Figures 4, 5). Antibiotics alone are not considered immune activation conditions for termites, so simple antibiotic treatment will not cause immune response in termites. Calcium (Ca^2+^) is a second messenger that participates in the modulation of various biological processes, including cell survival, cell proliferation, apoptosis, and the immune response (Kong et al., 2021). There is increasing evidence that Ca^2+^ signaling is beneficial for JAK-STAT activation induced by different stimuli and that it plays a vital role in regulating the activation of NF-κB to facilitate gene transcription (Wang et al., 2008; Liu et al., 2016). In addition, we found that the expression of genes related to melanogenesis, which is involved in the innate immunity of insects (Koike and Yamasaki, 2020), changed dramatically in the IAA treatment group (Figure 4). Our GO enrichment analysis also showed a lack of DEGs involved in direct immune pathways (Figure 5). Rather than directly impacting immune pathway gene expression, gut bacteria may contribute to host immunity through other means, such as by affecting host signal transduction and melanogenesis or by changing intestinal oxygen levels at the time of pathogen infestation (Nappi and Vass, 1993; Nappi et al., 2009).

Using qRT-PCR analysis, we measured the gene expression changes of the top 20 most significant DEGs identified from our transcriptomic study (Figure 6). We found that antibiotic treatment did not affect the expression of chitinase in the host (Figure 6D). At the same time, expression of the reverse transcription-related genes *RT-like 1*, *RT-like 2* and *RT-like 3* in the intestinal tract were decreased after antibiotic treatment (Figures 6A,E). Studies have shown that intestinal microbial imbalance can cause cell cancerization and changes in DNA levels, especially in adverse environments (Sobhani et al., 2019). It can be speculated that the presence of pathogenic bacteria does not affect the reverse transcription process of termites, but that the imbalance of intestinal microorganisms leads to this change. Expression of the apolipoprotein gene *APOA2* was upregulated in MI termites but downregulated in IAA termites (Figure 6B). Apolipoproteins participate in the innate immune regulation of insects, demonstrating antibacterial activity and cooperation with antimicrobial peptides (Rahman et al., 2006; Hanada et al., 2011; Feingold and Grunfeld, 2012; Kamareddine et al., 2016). The pattern of expression that was observed in MI and IAA termites suggests that the balance of intestinal microorganisms is necessary for termites to resist pathogen invasion. Interestingly, genes associated with calcium signal pathway were highly enriched in our KEGG analysis, and calpain (Figure 6B) is related to apoptosis and cell division (Shi et al., 2022). Finally, *haptoglobin* (Figure 6B) contains trypsin-like serine protease, which is also related to immunity (Pászti-Gere et al., 2021; Yang et al., 2021). Combined with the above results, we speculate that antibiotic treatment slows down the apoptosis of host cells and increases the expression of apolipoprotein on the surface of cell membranes.

We also measured *HSP70* and *ATP-syn* gene expression in all four treatment groups (Figure 6E). These genes are involved in physiological stress processes, aiding primarily in the folding and stretching of newly synthesized proteins and in the repair of misfolded proteins (Fernández-Fernández and Valpuesta, 2018). Under the stimulation of fungal infection, the expressions of these genes increased. It is speculated that when insects encounter pathogens, *HSP70* is upregulated to protect against cellular injury (Tang et al., 2012). Expression of *ATP-syn* was increased in MI termites, while decreased in IAA termites. We speculate that *M. robertsii* infection led to an increase of protein synthesis in the host, resulting in a large amount of ATP production and consumption. Downregulation of *ATP-syn* in the IAA group suggests that intestinal symbionts play a role in host ATP synthesis during pathogen invasion (Peterson and Scharf, 2016a).

## 5 Conclusion

In this study, termites exposed to antibiotics followed by a fungal pathogen showed significant changes in gene expression in comparison to those challenged exclusively by one factor. Notably, these changes affected genes associated with calcium signaling pathway and melanization process. These results suggest that intestinal symbionts may play a buffering role when the insect host encounters foreign pathogens, stimulating the expression of immune related genes, such as apolipoprotein. Symbionts may also affect the protein synthesis process and energy supply of the host through *HSP70* and *ATP synthesis*. The gut microbiota provides essential health benefits to its host, particularly by maintaining homeostasis. Disruption of such homeostatic balance can lead to significant adverse impacts, including diseases and lethality. Given that gut microbiota and host immune system have coevolved to maintain homeostasis, genetic underpinnings govern the dialogues between the two is one of the emerging questions warrant immediate attention.

## Data Availability

The datasets presented in this study can be found in online repositories. The names of the repository/repositories and accession number(s) can be found below: https://www.ncbi.nlm.nih.gov/, PRJNA926100.

## References

[B1] Aida C. Irene R. Claudia D. Michela M. Paolo R. Patrizia S. (2013). Interactions between asaia, plasmodium and Anopheles: New insights into mosquito symbiosis and implications in malaria symbiotic control. Parasites Vectors 6 (1), 13. 10.1186/1756-3305-6-182 23777746 PMC3708832

[B2] Altschul S. F. Madden T. L. Schäffer A. A. Zhang J. Zhang Z. Miller W. (1997). Gapped BLAST and PSI-BLAST: A new generation of protein database search programs. Nucleic Acids Res. 25 (17), 3389–3402. 10.1093/nar/25.17.3389 9254694 PMC146917

[B3] Avulova S. Rosengaus R. B. (2011). Losing the battle against fungal infection: Suppression of termite immune defenses during mycosis. J. Insect Physiol. 57 (7), 966–971. 10.1016/j.jinsphys.2011.04.009 21530532

[B4] Bai L. Wang L. Vega-Rodriguez J. Wang G. Wang S. (2019). A gut symbiotic bacterium *Serratia marcescens* renders mosquito resistance to plasmodium infection through activation of mosquito immune responses. Front. Microbiol. 10, 1580. 10.3389/fmicb.2019.01580 31379768 PMC6657657

[B5] Buchon N. Broderick N. A. Chakrabarti S. Lemaitre B. (2009). Invasive and indigenous microbiota impact intestinal stem cell activity through multiple pathways in Drosophila. Genes Dev. 23 (19), 2333–2344. 10.1101/gad.1827009 19797770 PMC2758745

[B6] Chen S. Zhou Y. Chen Y. Gu J. (2018). fastp: an ultra-fast all-in-one FASTQ preprocessor. Bioinformatics 34 (17), 1884–i890. 10.1093/bioinformatics/bty560 30423086 PMC6129281

[B7] Cheng D. Guo Z. Riegler M. Xi Z. Liang G. Xu Y. (2017). Gut symbiont enhances insecticide resistance in a significant pest, the oriental fruit fly Bactrocera dorsalis (Hendel). Microbiome 5 (1), 13. 10.1186/s40168-017-0236-z 28143582 PMC5286733

[B8] Chouvenc T. Su N. Y. Robert A. (2009). Inhibition of Metarhizium anisopliae in the alimentary tract of the eastern subterranean termite Reticulitermes flavipes. J. Invertebr. Pathol. 101 (2), 130–136. 10.1016/j.jip.2009.04.005 19426734

[B9] Cremer S. Pull C. D. Furst M. A. (2018). Social immunity: Emergence and evolution of colony-level disease protection. Annu. Rev. Entomol. 63, 105–123. 10.1146/annurev-ento-020117-043110 28945976

[B10] Davidson N. M. Oshlack A. (2014). Corset: Enabling differential gene expression analysis for de novo assembled transcriptomes. Genome Biol. 15 (7), 410. 10.1186/s13059-014-0410-6 25063469 PMC4165373

[B11] Deng J. Xu W. Lv G. Yuan H. Zhang Q. H. Wickham J. D. (2022). Associated bacteria of a pine sawyer beetle confer resistance to entomopathogenic fungi via fungal growth inhibition. Environ. Microbiome 17 (1), 47. 10.1186/s40793-022-00443-z 36085246 PMC9463743

[B12] Dong Y. Manfredini F. Dimopoulos G. (2009). Implication of the mosquito midgut microbiota in the defense against malaria parasites. PLoS Pathog. 5 (5), e1000423. 10.1371/journal.ppat.1000423 19424427 PMC2673032

[B13] Douglas A. E. (2018). The Drosophila model for microbiome research. Lab. Anim. (NY) 47 (6), 157–164. 10.1038/s41684-018-0065-0 29795158 PMC6586217

[B14] El-Gebali S. Mistry J. Bateman A. Eddy S. R. Luciani A. Potter S. C. (2019). The Pfam protein families database in 2019. Nucleic Acids Res. 47 (D1), D427–d432. 10.1093/nar/gky995 30357350 PMC6324024

[B15] Feingold K. R. Grunfeld C. (2012). Lipids: A key player in the battle between the host and microorganisms. J. Lipid Res. 53 (12), 2487–2489. 10.1194/jlr.E033407 23075464 PMC3494250

[B16] Fernández-Fernández M. R. Valpuesta J. M. (2018). Hsp70 chaperone: A master player in protein homeostasis. F1000Res 7. 10.12688/f1000research.15528.1 PMC6148205 30338057

[B17] Gao H. Bai L. Jiang Y. Huang W. Wang L. Li S. (2021). A natural symbiotic bacterium drives mosquito refractoriness to Plasmodium infection via secretion of an antimalarial lipase. Nat. Microbiol. 6 (6), 806–817. 10.1038/s41564-021-00899-8 33958765 PMC9793891

[B18] Garcia-Robles I. De Loma J. Capilla M. Roger I. Boix-Montesinos P. Carrion P. (2020). Proteomic insights into the immune response of the Colorado potato beetle larvae challenged with Bacillus thuringiensis. Dev. Comp. Immunol. 104, 103525. 10.1016/j.dci.2019.103525 31655128

[B19] Grabherr M. G. Haas B. J. Yassour M. Levin J. Z. Thompson D. A. Amit I. (2011). Full-length transcriptome assembly from RNA-Seq data without a reference genome. Nat. Biotechnol. 29 (7), 644–652. 10.1038/nbt.1883 21572440 PMC3571712

[B20] Gravitz L. (2012). Microbiome: The critters within. Nature 485 (7398), S12–S13. 10.1038/485s12a 22616099

[B21] Hanada Y. Sekimizu K. Kaito C. (2011). Silkworm apolipophorin protein inhibits *Staphylococcus aureus* virulence. J. Biol. Chem. 286 (45), 39360–39369. 10.1074/jbc.M111.278416 21937431 PMC3234760

[B22] He S. Johnston P. R. Kuropka B. Lokatis S. Weise C. Plarre R. (2018). Termite soldiers contribute to social immunity by synthesizing potent oral secretions. Insect Mol. Biol. 27 (5), 564–576. 10.1111/imb.12499 29663551

[B23] Hong M. Hwang D. Cho S. (2018). Hemocyte morphology and cellular immune response in termite (Reticulitermes speratus). J. Insect Sci. 18 (2), 46. 10.1093/jisesa/iey039 29718507 PMC5917771

[B24] Hu H. da Costa R. R. Pilgaard B. Schiott M. Lange L. Poulsen M. (2019). Fungiculture in termites is associated with a mycolytic gut bacterial community. mSphere 4 (3), 001655–e219. 10.1128/mSphere.00165-19 PMC6520439 31092601

[B25] Kamareddine L. Nakhleh J. Osta M. A. (2016). Functional interaction between apolipophorins and complement regulate the mosquito immune response to systemic infections. J. Innate Immun. 8 (3), 314–326. 10.1159/000443883 26950600 PMC6738827

[B26] Kanehisa M. Araki M. Goto S. Hattori M. Hirakawa M. Itoh M. (2008). KEGG for linking genomes to life and the environment. Nucleic Acids Res. 36, D480–D484. 10.1093/nar/gkm882 18077471 PMC2238879

[B27] Koike S. Yamasaki K. (2020). Melanogenesis connection with innate immunity and toll-like receptors. Int. J. Mol. Sci. 21 (24), 9769. 10.3390/ijms21249769 33371432 PMC7767451

[B28] Kong F. You H. Zheng K. Tang R. Zheng C. (2021). The crosstalk between pattern-recognition receptor signaling and calcium signaling. Int. J. Biol. Macromol. 192, 745–756. 10.1016/j.ijbiomac.2021.10.014 34634335

[B29] Li B. Dewey C. N. (2011). RSEM: Accurate transcript quantification from RNA-seq data with or without a reference genome. BMC Bioinforma. 12, 323. 10.1186/1471-2105-12-323 PMC3163565 21816040

[B30] Li F. Li M. Zhu Q. Mao T. Dai M. Ye W. (2021). Imbalance of intestinal microbial homeostasis caused by acetamiprid is detrimental to resistance to pathogenic bacteria in *Bombyx mori* . Environ. Pollut. 289, 117866. 10.1016/j.envpol.2021.117866 34343750

[B31] Liu N. Zhang L. Zhou H. Zhang M. Yan X. Wang Q. (2013). Metagenomic insights into metabolic capacities of the gut microbiota in a fungus-cultivating termite (Odontotermes yunnanensis). PLoS One 8 (7), e69184. 10.1371/journal.pone.0069184 23874908 PMC3714238

[B32] Liu L. Li G. Sun P. Lei C. Huang Q. (2015). Experimental verification and molecular basis of active immunization against fungal pathogens in termites. Sci. Rep. 5, 15106. 10.1038/srep15106 26458743 PMC4602225

[B33] Liu X. Berry C. T. Ruthel G. Madara J. J. MacGillivray K. Gray C. M. (2016). T cell receptor-induced nuclear factor κB (NF-κB) signaling and transcriptional activation are regulated by STIM1-and orai1-mediated calcium entry. J. Biol. Chem. 291 (16), 8440–8452. 10.1074/jbc.M115.713008 26826124 PMC4861418

[B34] Love M. I. Huber W. Anders S. (2014). Moderated estimation of fold change and dispersion for RNA-seq data with DESeq2. Genome Biol. 15 (12), 550. 10.1186/s13059-014-0550-8 25516281 PMC4302049

[B35] Mao X. Cai T. Olyarchuk J. G. Wei L. (2005). Automated genome annotation and pathway identification using the KEGG Orthology (KO) as a controlled vocabulary. Bioinformatics 21 (19), 3787–3793. 10.1093/bioinformatics/bti430 15817693

[B36] Moriya Y. Itoh M. Okuda S. Yoshizawa A. C. Kanehisa M. (2007). KAAS: An automatic genome annotation and pathway reconstruction server. Nucleic Acids Res. 35, W182–W185. 10.1093/nar/gkm321 17526522 PMC1933193

[B37] Nappi A. J. Vass E. (1993). Melanogenesis and the generation of cytotoxic molecules during insect cellular immune reactions. Pigment. Cell Res. 6 (3), 117–126. 10.1111/j.1600-0749.1993.tb00590.x 8234196

[B38] Nappi A. Poirié M. Carton Y. (2009). The role of melanization and cytotoxic by-products in the cellular immune responses of Drosophila against parasitic wasps. Adv. Parasitol. 70, 99–121. 10.1016/s0065-308x(09)70004-1 19773068

[B39] Pászti-Gere E. Pomothy J. Jerzsele Á. Pilgram O. Steinmetzer T. (2021). Exposure of human intestinal epithelial cells and primary human hepatocytes to trypsin-like serine protease inhibitors with potential antiviral effect. J. Enzyme Inhib. Med. Chem. 36 (1), 659–668. 10.1080/14756366.2021.1886093 33641565 PMC7928042

[B40] Peterson B. F. Scharf M. E. (2016a). Lower termite associations with microbes: Synergy, protection, and interplay. Front. Microbiol. 7, 422. 10.3389/fmicb.2016.00422 27092110 PMC4824777

[B41] Peterson B. F. Scharf M. E. (2016b). Metatranscriptome analysis reveals bacterial symbiont contributions to lower termite physiology and potential immune functions. BMC Genomics 17 (1), 772. 10.1186/s12864-016-3126-z 27716053 PMC5045658

[B42] Peterson B. F. Stewart H. L. Scharf M. E. (2015). Quantification of symbiotic contributions to lower termite lignocellulose digestion using antimicrobial treatments. Insect Biochem. Mol. Biol. 59, 80–88. 10.1016/j.ibmb.2015.02.009 25724277

[B43] Rahman M. M. Ma G. Roberts H. L. Schmidt O. (2006). Cell-free immune reactions in insects. J. Insect Physiol. 52 (7), 754–762. 10.1016/j.jinsphys.2006.04.003 16753175

[B44] Ramirez J. L. Souza-Neto J. Torres Cosme R. Rovira J. Ortiz A. Pascale J. M. (2012). Reciprocal tripartite interactions between the *Aedes aegypti* midgut microbiota, innate immune system and dengue virus influences vector competence. PLoS Negl. Trop. Dis. 6 (3), e1561. 10.1371/journal.pntd.0001561 22413032 PMC3295821

[B45] Rosengaus R. B. Schultheis K. F. Yalonetskaya A. Bulmer M. S. DuComb W. S. Benson R. W. (2014). Symbiont-derived beta-1,3-glucanases in a social insect: Mutualism beyond nutrition. Front. Microbiol. 5, 607. 10.3389/fmicb.2014.00607 25484878 PMC4240165

[B46] Shi H. Yu Y. Liu X. Yu Y. Li M. Wang Y. (2022). Inhibition of calpain reduces cell apoptosis by suppressing mitochondrial fission in acute viral myocarditis. Cell Biol. Toxicol. 38 (3), 487–504. 10.1007/s10565-021-09634-9 34365571 PMC9200683

[B47] Shu G. Ji Z. Wang Z. (2011). “Effect of kanamycin sulfate and gentamicin on growth of probiotics”, in: International Conference on Material Engineering, Architectural Engineering and Informatization (MEAEI).

[B48] Sobhani I. Bergsten E. Couffin S. Amiot A. Nebbad B. Barau C. (2019). Colorectal cancer-associated microbiota contributes to oncogenic epigenetic signatures. Proc. Natl. Acad. Sci. U. S. A. 116 (48), 24285–24295. 10.1073/pnas.1912129116 31712445 PMC6883805

[B49] Tang T. Wu C. Li J. Ren G. Huang D. Liu F. (2012). Stress-induced HSP70 from *Musca domestica* plays a functionally significant role in the immune system. J. Insect Physiol. 58 (9), 1226–1234. 10.1016/j.jinsphys.2012.06.007 22750549

[B50] Vikram S. Arneodo J. D. Calcagno J. Ortiz M. Mon M. L. Etcheverry C. (2021). Diversity structure of the microbial communities in the guts of four neotropical termite species. PeerJ 9, e10959. 10.7717/peerj.10959 33868801 PMC8035897

[B51] Wang L. Tassiulas I. Park-Min K. H. Reid A. C. Gil-Henn H. Schlessinger J. (2008). Tuning' of type I interferon-induced Jak-STAT1 signaling by calcium-dependent kinases in macrophages. Nat. Immunol. 9 (2), 186–193. 10.1038/ni1548 18084294

[B52] Wang L. Feng Z. Wang X. Wang X. Zhang X. (2010). DEGseq: an R package for identifying differentially expressed genes from RNA-seq data. Bioinformatics 26 (1), 136–138. 10.1093/bioinformatics/btp612 19855105

[B53] Wei G. Lai Y. Wang G. Chen H. Li F. Wang S. (2017). Insect pathogenic fungus interacts with the gut microbiota to accelerate mosquito mortality. Proc. Natl. Acad. Sci. U. S. A. 114 (23), 5994–5999. 10.1073/pnas.1703546114 28533370 PMC5468619

[B54] Xi Z. Ramirez J. L. Dimopoulos G. (2008). The *Aedes aegypti* toll pathway controls dengue virus infection. PLoS Pathog. 4 (7), e1000098. 10.1371/journal.ppat.1000098 18604274 PMC2435278

[B55] Xiong W. (2022). Intestinal microbiota in various animals. Integr. Zool. 17 (3), 331–332. 10.1111/1749-4877.12633 35182033

[B56] Yang H. Ji T. Xiong H. Zhang Y. Wei W. (2021). A trypsin-like serine protease domain of masquerade gene in crayfish *Procambarus clarkii* could activate prophenoloxidase and inhibit bacterial growth. Dev. Comp. Immunol. 117, 103980. 10.1016/j.dci.2020.103980 33340591

[B57] Young M. D. Wakefield M. J. Smyth G. K. Oshlack A. (2010). Gene ontology analysis for RNA-seq: Accounting for selection bias. Genome Biol. 11 (2), R14. 10.1186/gb-2010-11-2-r14 20132535 PMC2872874

[B58] Zhou F. Xu L. Wu X. Zhao X. Liu M. Zhang X. (2020). Symbiotic bacterium-derived organic acids protect *Delia antiqua* larvae from entomopathogenic fungal infection. mSystems 5 (6), 007788–e820. 10.1128/mSystems.00778-20 PMC7677000 33203688

