# Supplementary material for: Transcriptomic dissection of termite gut microbiota following entomopathogenic fungal infection
Source: Front Physiol. 2023 Apr 21;14:1194370. doi: 10.3389/fphys.2023.1194370 (PMC10161392; doi:10.3389/fphys.2023.1194370)
Supplement: Supplementary file 1 [file DataSheet1.docx]

Supplementary Material

Transcriptomic dissection of termite gut microbiota following entomopathogenic fungal infection

**Ya-ling Tang^1, 2^, Yun-hui Kong^1^, Sheng Qin^1, 3^, Austin Merchant^4^, Ji-zhe Shi^4^, Xu-guo Zhou^4*^, Mu-wang Li^1, 3*^ and Qian Wang^2,*^**

**^1^Jiangsu Key Laboratory of Sericultural Biology and Biotechnology, School of Biotechnology, Jiangsu University of Science and Technology, Zhenjiang, Jiangsu Province, China**

**^2^Shanghai First Maternity and Infant Hospital, Tongji University School of Medicine, Shanghai, China**

**^3^Key Laboratory of Silkworm and Mulberry Genetic Improvement, Ministry of Agriculture and Rural Affairs, Sericultural Research Institute, Chinese Academy of Agricultural Science, Zhenjiang, Jiangsu Province, China**

**^4^Department of Entomology, University of Kentucky, Lexington, KY, United States**

*** Correspondence:**Xu-guo Zhou: [xuguozhou@uky.edu](mailto:xuguozhou@uky.edu)

Mu-wang Li: [mwli@just.edu.cn](mailto:mwli@just.edu.cn)

Qian Wang: wangqian2017@tongji.edu.cn

**Supplementary Table 1.** Frequency distribution of splicing length

| Transcript length interval | 300-500bp | 500-1kbp | 1k-2kbp | >2kbp | Total |
| --- | --- | --- | --- | --- | --- |
| Number of transcripts | 49102 | 36016 | 22016 | 35397 | 142531 |
| Number of Unigenes | 29428 | 21812 | 10087 | 12281 | 73608 |

**Supplementary Table 2.** Splice length distribution

| Type | Min length | Mean length | Median length | Max length | N50 | N90 | Total nucleotide |
| --- | --- | --- | --- | --- | --- | --- | --- |
| Transcript | 301 | 1684 | 721 | 53895 | 3655 | 576 | 240083960 |
| Unigene | 301 | 1310 | 595 | 53895 | 2746 | 464 | 96392244 |

**Supplementary Table 3.** Proportion of different query sequence length with annotations in the public databases

| Database | Number of Unigenes | Percentage |
| --- | --- | --- |
| Annotated in NR | 27768 | 37.72 |
| Annotated in NT | 16153 | 21.94 |
| Annotated in KO | 9583 | 13.01 |
| Annotated in SwissProt | 16345 | 22.2 |
| Annotated in PFAM | 23052 | 31.31 |
| Annotated in GO | 23049 | 31.31 |
| Annotated in KOG | 8594 | 11.67 |
| Annotated in all Databases | 3729 | 5.06 |
| Annotated in at least one Database | 38999 | 52.98 |
| Total Unigenes | 73608 | 100 |

**Supplementary Table 4.** Primers used in this study

| Gene Names | Forward Primer (5’-3’) | Reverse Primer (5’-3’) |
| --- | --- | --- |
| 7668.SPU_003371-tr | AGATGTGCCTCCTGCGACCTT | GCCTCACGATGTCTGCCTCCTA |
| 103372.F4WUE8 | AACGGTGAACGCCATTCCATCG | CGCACAAGCTGACAGACGAACA |
| 6326.BUX.s01206.1 | GAGCACTTCAGGAGCCGTCTTG | AGAAGCCAGCCGACCAGCAT |
| 121225.PHUM165840-PA | CCCTCCACATGGCACACAACTG | CCTCAGCACGCAACATGGCAA |
| 9541.XP_005592466.1 | GCTGTGGTGTGGCTGAGTATGG | ATTCCCGTCCACTTTGCCCTCT |
| 10224.XP_006811278.1 | CCACCAGAGACCCACCCGATTT | GCCAAGTGACAACCTCCGTTCC |
| 136037.KDR12186 | AGCCAGGCACACCATCACTGT | GCCGACTGTTCCACCCTCATTC |
| 121225.PHUM363230-PA | TGGCTGGCTGGCTGTGAGAA | GGCTACCGGATTGCACAACTCC |
| 61622.XP_010365434.1 | CGGTGACTGACTACGGCAAGGA | ACTGGGTGGCAGGTTGTGTTCT |
| 7739.XP_002606072.1 | CGTGTTGGATTCTGGTGATGGT | TCGGCTGTGGTTCTGAATGTGT |
| 9544.ENSMMUP00000031379 | AAGTGTCAGTACCAAGCGGCAG | TACCACAGGCTTCCACGGACTC |
| 7425.NV17477-PA | ATGGACTCGTGGAGCGGTTACA | GGTGGCTGGTGTCAGGAACTCT |
| 136037.KDR14744 | AGTTCCATCTGCGGACCACGAA | AGCGAAGTGCCAGTGCCAGTA |
| 28377.ENSACAP00000023447 | GGGTAAGACAAGGCTGTCCACT | GTTGGCACTAACTTCGCTGACC |
| 9778.XP_004378770.1 | CGACGGCTCAGTGGAAACAGT | TCCGACTTCAGCACATCACCAA |
| 136037.KDR23754 | ATCGACCAGCGAGCACTACACA | CACTGAACAGCGGGCAGGAAAT |
| 136037.KDR09026 | CAACACGCTGTCAGGCACGAT | CAGAACACAAGCAGGACGCAGA |
| 136037.KDR16219 | ACGCACTCCAACGCACACTG | CGAGAGCACCAGGCGGTTATTG |
| 136037.KDR23635 | TGACCGAGCAGCGAAGGAAGT | GCAGTGGCACCTCCAACTCTTC |
| 136037.KDR14276 | CCAATACCGCTGCTTGCTCCTG | ACTGGGTGCCTGTGGACGAAA |
| 16s | ACTCCTACGGGAGGCAGCAG | ATTACCGCGGCTGCTGG |
| *β-actin* | CTCTTCCAGCCTTCCTTCCT | CTTCTGCATCCTGTCAGCAA |

Notes: Primers1-20 for validation of differentially expressed transcript; 16s was used in testify the change of total bacteria in termite hindgut; *β-actin* was the reference gene.

**Supplementary Table 5.** Blast result of the top 20 DEGs in transcriptome

| Gene Names | Blast Name | e-value | Names in text |
| --- | --- | --- | --- |
| *7668.SPU_003371-tr* | - | - | *RT-like-1* |
| *103372.F4WUE8* | Uncharacterized | 0.00018 | *MCT* |
| *6326.BUX.s01206.1* | - | - | *Vir-G2* |
| *121225.PHUM165840-PA* | Coiled-coil domain-containing protein 39 | 5.42E-112 | *Coiled-coil 39* |
| *9541.XP_005592466.1* | Haptoglobin protein | 2.15E-269 | *Hap* |
| *10224.XP_006811278.1* | - | - | *RT-nLTR* |
| *136037.KDR12186* | - | - | *Us7* |
| *121225.PHUM363230-PA* | Calpain-5-like | 5.78E-64 | *CAPN5-like* |
| *61622.XP_010365434.1* | Apolipoprotein A2 | 4.35E-61 | *Apo A2* |
| *7739.XP_002606072.1* | Actin-5c | 4.96E-235 | *Hsp70* |
| *9544.ENSMMUP00000031379* | predicted cytochrone oxidase subunit2-like | 6.67E-30 | *ATP-syn* |
| *7425.NV17477-PA* | Uncharacterized | 1.17E-41 | *RT-like-2* |
| *136037.KDR14744* | Unknown function | 6.36E-85 | *NADB* |
| *28377.ENSACAP00000023447* | Uncharacterized | 5.91E-19 | *RT-like-3* |
| *9778.XP_004378770.1* | - | - | *PKC* |
| *136037.KDR23754* | Chitinase 5 | 9.78E-292 | *Chitinase 5* |
| *136037.KDR09026* | sodium-coupled monocarboxylate transporter 1 | 7.39E-222 | *Sot1* |
| *136037.KDR16219* | sodium/potassium/calcium exchanger 4-like | 5.96E-217 | *Sot4-like* |
| *136037.KDR23635* | Dynein heavy chain 7 axonemal | 8.29E-45 | *DHC7A* |
| *136037.KDR14276* | Probable multidrug resistance-associated protein lethal (2) | 1.3E-106 | *MRAP* |

Notes: “-”: means no result. Annotations were based on their PFAMs or blast results.

**Supplementary Figure S1.** Histogram of Gene Ontology (GO) classification.

**
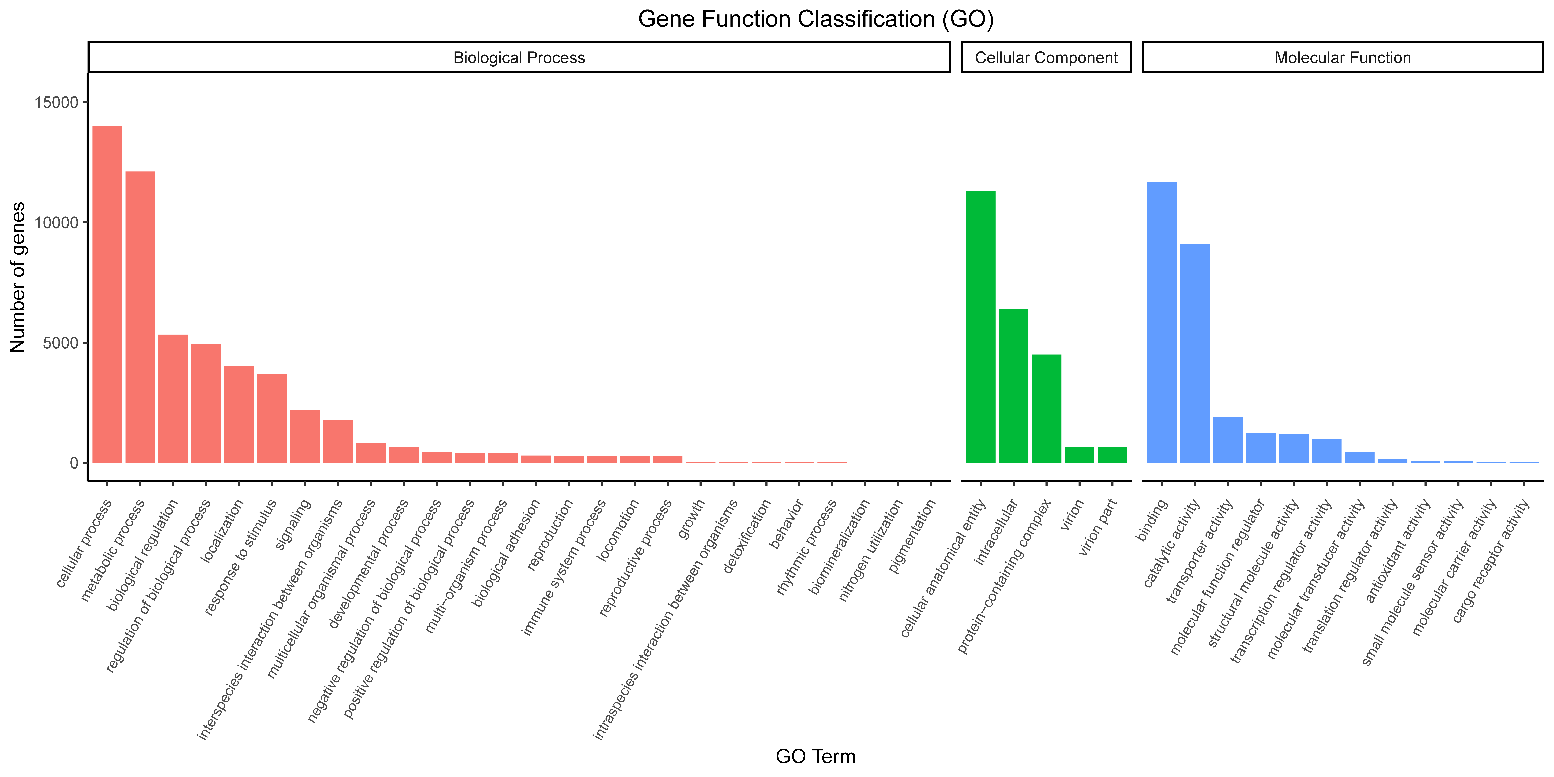
**

The results were summarized into three categories: Biological process, Cellular component and Molecular function.

**Supplementary Figure S2.** KOG categories for the Odontotermes formosanus workers’ gut transcriptome.

**
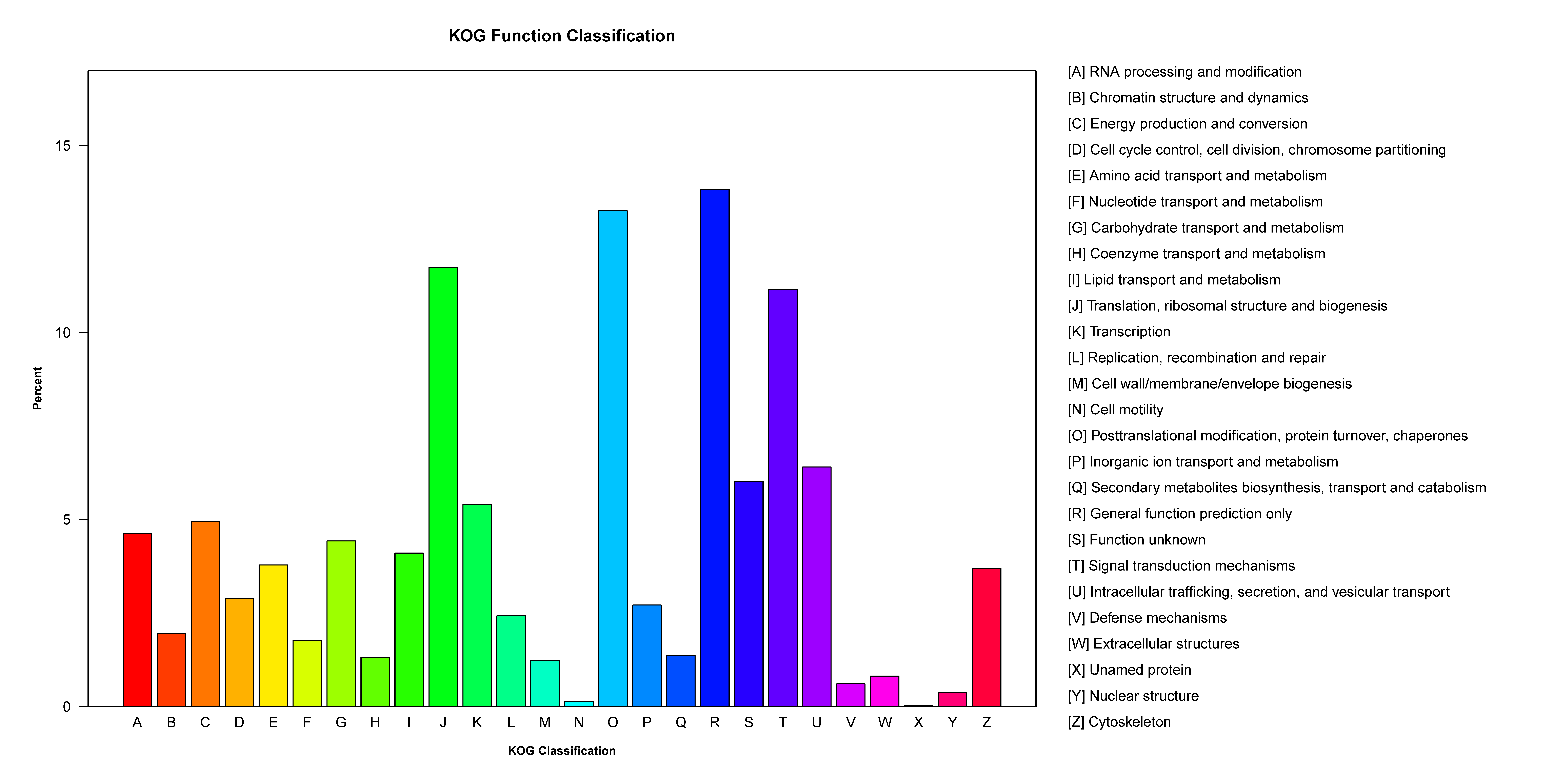
**

**Supplementary Figure S3.** Histogram of differential expression genes between four groups.

**
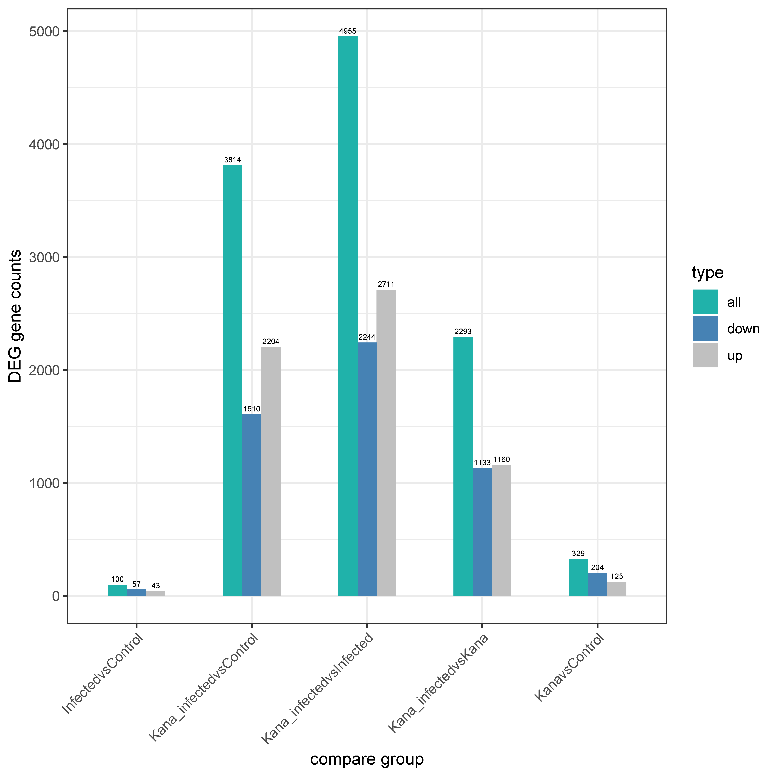
**

**Supplementary Figure S4.** KEGG Enrichment of IAA: CT and MI: AT comparison in transcriptome data.

**
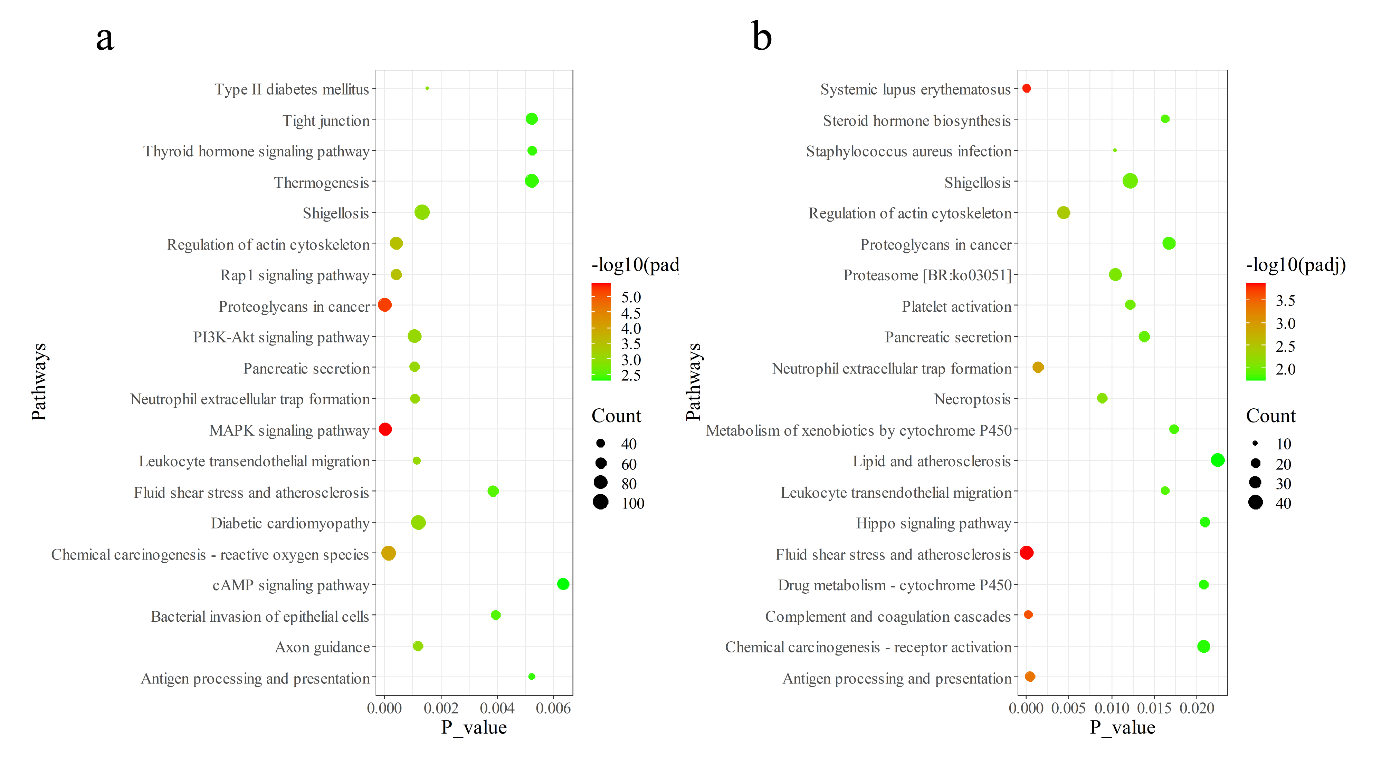
**

**a**: KEGG pathway enrichment of IAA: CT comparison DEGs. **b**: KEGG pathway enrichment of MI: AT comparison DEGs
